# Circ_0138960 contributes to lipopolysaccharide‐induced periodontal ligament cell dysfunction

**DOI:** 10.1002/iid3.732

**Published:** 2022-11-07

**Authors:** Shuangshuang Li, Huilin Xu, Yuanyuan Li, Ruijing Li

**Affiliations:** ^1^ Department of Stomatology Dongying Shengli Oilfield Central Hospital Dongying City Shandong Province China

**Keywords:** circ_0138960, HDAC6, lipopolysaccharide, miR‐518a‐5p, periodontal ligament cells, periodontitis

## Abstract

**Background:**

Periodontitis is a common oral inflammatory disease, and lipopolysaccharide (LPS) is a key risk factor in periodontitis pathology. Here, we used LPS‐induced periodontal ligament cells (PDLCs) to explore the molecular mechanism of periodontitis.

**Methods:**

Cell viability, proliferation, and apoptosis were analyzed by Cell Counting Kit‐8, 5‐ethynyl‐20‐deoxyuridine (EDU), and flow cytometry assays, respectively. Apart from that, their targeting relationship was validated using dual‐luciferase reporter and RNA‐pull down.

**Results:**

Circular RNA_0138960 (circ_0138960) was notably upregulated in periodontitis sufferers (*p* < .001) and LPS‐disposed PDLCs (*p* < .05). LPS exposure dampened PDLC proliferation, and promoted apoptosis and inflammation (*p* < .05). Circ_0138960 acted as a microRNA sponge for miR‐518a‐5p to affect histone deacetylase 6 (HDAC6) expression. Circ_0138960 absence‐mediated protective effects in LPS‐induced PDLCs were largely abrogated via silencing miR‐518a‐5p or HDAC6 overexpression (*p* < .05).

**Conclusion:**

Circ_0138960 promoted LPS‐induced dysfunction in PDLCs by targeting miR‐518a‐5p/HDAC6 axis, which provided novel potential therapeutic targets for periodontitis.

## Graphical abstract

Please designate which figure should be used for the graphical table of contents and considered for the publication cover.

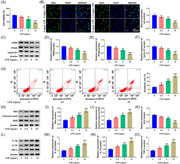

## 1 INTRODUCTION

As a progressive disease of periodontal tissue, Periodontitis is ascribed to the accumulation of subgingival pathogens.^1^ The pathology of periodontitis is associated with environmental factors (bad living habits and smoking) and genetic factors.^2^ Current research revealed that clarification on the mechanisms involving periodontitis is of great significance for the prevention of periodontitis. Lipopolysaccharide (LPS) is highly toxic to the periodontal ligament and can induce cellular dysfunction, thus contributing to periodontitis progression.^3^ Hence, LPS‐induced periodontal ligament cells (PDLCs) are generally utilized as periodontitis cell models in vitro.^4^, ^5^

Circular RNAs (circRNAs) with conserved stable loop structures highlighted their potential as biomarkers for human diseases.^6^ CircRNAs play important regulatory roles in periodontitis pathology.^5^, ^7^ Circ_0081572 expression is downregulated in patients with periodontal disease, and circ_0081572 overexpression attenuates LPS‐induced dysfunction in PDLCs.^8^ Here, we focus on an abnormally upregulated circRNA, circ_0138960, in patients with periodontal disease,^9^ but its biological role and mechanism in periodontitis pathology remain to be clarified.

Recent reports have described that circRNAs act as microRNA (miRNA) sponges to indirectly modulate gene expression, thus affecting cell behaviors.^10^, ^11^ This study first investigated the role of circ_0138960 in LPS‐triggered PDLC dysfunction. Subsequently, we explored the downstream miRNA/Messenger RNA (mRNA) axis of circ_0138960 to uncover its working mechanism in periodontitis pathology.

## 2 MATERIALS AND METHODS

### 2.1 Gingival tissues

After being signed written informed consent, human gingival tissues were collected from patients with periodontal disease (*n* = 37) or from normal controls (*n* = 25) who received gingivectomy during orthodontic treatment at Dongying Shengli Oilfield Central Hospital. Diagnostic criteria of periodontitis were presented as follows: (1) redness and swelling of the gingiva on the surface of the periodontal pocket or bleeding after probing; (2) probe depth > 3 mm and attachment loss > 1 mm; (3) X‐ray showed horizontal or vertical resorption of alveolar bone. Exclusion criteria included patients with systemic diseases and antibiotic intake within 1 month before the surgery. Approval to perform this project was acquired from the Ethics Committee of Dongying Shengli Oilfield Central Hospital. NO. S2020623. Table 1 displayed the clinicopathologic characteristics of periodontitis sufferers.

**Table 1:** Correlation between circ_0138960 expression and clinicopathologic characteristics in patients with periodontal disease (*N* = 37)

Characteristics	Number	circ_0138960 expression		*p* Value	Odds ratio
		Low (*N* = 18)	High (*N* = 19)		
Age (years)				.8584	1.125
<65	20 (54%)	10	10		
≥65	17 (46%)	8	9		
Sex				.6301	1.375
Male	16 (43%)	9	8		
Female	21 (57%)	9	11		
Order of severity				.0029	11.00
Slight + medium	24 (65%)	16	8		
Severe	13 (35%)	2	11		
Grade of periodontitis				.0220	5.556
Level 0 + 1	24 (65%)	15	9		
Level 2	13 (35%)	3	10		

*Note*: Level 0, individuals with a healthy periodontium and up to one proximal site with loss of attachment ≥ 3 mm; Level 1, presence of proximal attachment and loss ≥ 3 mm in ≥2 nonadjacent teeth; Level 2, presence of proximal attachment loss ≥ 5 mm in ≥30% of teeth.

### 2.2 Reverse transcription‐quantitative polymerase chain reaction (RT‐qPCR)

RNA samples were isolated from gingival tissues and PDLCs with Trizol reagent (Invitrogen). RNA was reversely transcribed to complementary DNA (cDNA) using an M‐MLV kit (Invitrogen) and miRNA cDNA Synthesis Kit (GeneCopoeia). Then, qPCR was carried out using the Power SYBR‐Green PCR master mix (Applied Biosystems). After normalization with β‐actin or U6, the obtained results were processed using the $2^{-\Delta \Delta C_{t}}$. All primers were shown in Table 2.

**Table 2:** Primer sequences used for qPCR

Name	Primers for qPCR (5'−3')	
hsa_circ_0138960	Forward	AAGGAGACCACTGAGGAATCG
	Reverse	CAGCAAAGGAATACTGAGAGGC
HDAC6	Forward	CCACACTGGGGTTCCCATAG
	Reverse	AGAAAATACTGGCCGTCGCC
miR‐518a‐5p	Forward	GCCGAGCTGCAAAGGGAAG
	Reverse	CTCAACTGGTGTCGTGGAG
β‐actin	Forward	CTTCGCGGGCGACGAT
	Reverse	CCACATAGGAATCCTTCTGACC
U6	Forward	CTCGCTTCGGCAGCACA
	Reverse	AACGCTTCACGAATTTGCGT

Abbreviation: HDAC6, histone deacetylase 6.

### 2.3 Cell isolation, culture, and identification

Teeth were collected from premolar teeth extracted from healthy patients (mean age: 14) for orthodontic treatment. Pretreated extracted teeth need to be disinfected with 1% iodine and 72% alcohol. After extraction, these samples from the middle third of the periodontal membrane root were washed and managed with α‐MEM. After being acquired from the root surface, PDL tissues at 37°C were incubated with the medium plus 1% penicillin/streptomycin. Meanwhile, the fourth to seventh generations of PDLCs were used for the following research. PDLCs were identified using the avidin‐biotin complex immunohistochemistry.

### 2.4 LPS treatment

PDLCs were stimulated with *Porphyromonas gingivalis* LPS (Sigma) at the dose of 2.5, 5, or 10 ng/μl for 12 h. Finally, PDLCs were treated with 10 ng/μl LPS for 12 h to induce the periodontitis‐like injury, which was used for further analysis.

### 2.5 Cell Counting Kit‐8 (CCK8) assay

2 × 10^3^ PDLCs were incubated with CCK8 solution (Sigma) for 4 h. The optical density at 450 nm was examined under a microplate reader (NYW‐96M; Nuoyawei Biotech).

### 2.6 5‐ethynyl‐20‐deoxyuridine (EDU) assay

PDLCs were incubated with 100 µl 50 μM EDU reagent (Ribobio) for 2 h at 37°C. Then, cells were immobilized with 4% paraformaldehyde for 30 min at 25°C and incubated with glycine for 4 h at 37°C. Subsequently, PDLCs were mixed with Apollo reaction cocktail and Triton X‐100 solution for 20 min. Then, the nucleus of PDLCs was stained with DAPI (Sigma) at 37°C. The fluorescence photographs of PDLCs were captured under a fluorescence microscope (Olympus).

### 2.7 Western blot assay

PDLCs were disrupted with RIPA reagent (Beyotime), and analyzed using a BCA kit (Thermo Fisher Scientific). Proteins were denatured in a boiling water bath for 10 min. After being subjected to 10% separating gel, samples were shifted to PVDF membranes, which then were mixed with primary antibodies (Abcam) at 4°C overnight: anti‐CDK6 (ab124821; 1:80000), anti‐proliferating cell nuclear antigen (PCNA) (ab29; 1:5000), anti‐Cyclin D1 (ab16663; 1:3000), anti‐Bax (ab32503; 1:8000), anti‐Cleaved‐casp3 (ab32042; 1:5000), anti‐Bcl2 (ab32124; 1:3000), anti‐IL‐1β (ab216995; 1:5000), anti‐IL‐18 (ab243091; 1:3000), anti‐TNF‐α (ab183218; 1:3000), anti‐histone deacetylase 6 (HDAC6) (ab133493; 1:20000), and anti‐β‐actin (ab8226; 1:20000). The visualization of signals was implemented according to an ECL Kit, after secondary antibody (ab6721/ab6789; 1:5000) incubation.

### 2.8 Flow cytometry

Annexin‐V‐FITC/PI dye liquor was prepared using 500 µl binding buffer, 10 µl Annexin‐V‐FITC, and 10 µl PI (BD Biosciences). PDLCs were incubated with the Annexin‐V‐FITC/PI dye liquor, away from light. Fifteen min later, the apoptotic rate was analyzed on the FC‐500 flow cytometer.

### 2.9 Cell transfection

In this study, PDLCs were further cultured into 96‐well plates overnight to reach 70%−0% confluence. Meanwhile, GenePharma and Ribobio offer circ_0138960‐specific siRNA (si‐circ_0138960), siRNA of HDAC6 (si‐HDAC6), miR‐518a‐5p mimics/inhibitor (miR‐518a‐5p/anti‐miR‐518a‐5p), HDAC6 plasmid (HDAC6), and controls (si‐NC, miR‐NC, anti‐miR‐NC, and plasmid cloning DNA[pcDNA]). Then, Lipofectamine™ 3000 reagent (Invitrogen) was utilized for transient transfection.

### 2.10 Establishment of circRNA/miRNA/mRNA axis

The interacted miRNAs of circ_0138960 and the interacted mRNAs of miR‐518a‐5p were predicted by Circinteractome (https://circinteractome.irp.nia.nih.gov) and targetscan (http://www.targetscan.org/vert_71) databases, respectively. All possible miRNA targets of circ_0138960 predicted by Circinteractome database were shown in Table 3.

**Table 3:** All possible miRNA targets of circ_0138960 predicted by Circinteractome database

Circ_0138960	Possible miRNA targets
	hsa‐miR‐1184
	hsa‐miR‐1205
	hsa‐miR‐1257
	hsa‐miR‐1286
	hsa‐miR‐1299
	hsa‐miR‐338‐3p
	hsa‐miR‐369‐5p
	hsa‐miR‐490‐5p
	hsa‐miR‐502‐5p
	hsa‐miR‐516b
	hsa‐miR‐518a‐5p
	hsa‐miR‐527
	hsa‐miR‐545
	hsa‐miR‐549
	hsa‐miR‐607
	hsa‐miR‐616
	hsa‐miR‐630
	hsa‐miR‐643
	hsa‐miR‐890

### 2.11 Dual‐luciferase reporter assay

A partial sequence of circ_0138960 or HDAC6 3' untranslated region (UTR) was amplified by qPCR. Meanwhile, their mutant sequence was synthesized via site‐directed mutation. The constructed reporter plasmids were termed circ_0138960^WT/MUT^ and HDAC6‐3'UTR^WT/MUT^. PDLCs were cotransfected with small RNAs and constructed plasmids, followed by analysis according to Dual‐Luciferase Reporter Assay kit (Promega) after transfection for 24 h.

### 2.12 RNA‐pull down assay

In short, Biotin‐labeled miR‐518a‐5p probe (Bio‐miR‐518a‐5p) and Bio‐miR‐NC were constructed by GenePharma. Cell lysates were simultaneously incubated with the probe and streptavidin agarose magnetic beads (Invitrogen). RNA enrichment was analyzed by RT‐qPCR.

Furthermore, cell extracts were simultaneously incubated with Biotin‐labeled circ_0138960 probe (circ_0138960 probe) or oligo probe (GenePharma) and streptavidin agarose magnetic beads (Invitrogen). miR‐1205, miR‐1299, miR‐518a‐5p, and miR‐527 expressions were examined using RT‐qPCR.

### 2.13 Statistical analysis

The mean between groups was compared using Student's *t*‐test or one‐way analysis of variance followed by Tukey's test. GraphPad Prism 7.0 software (GraphPad) was used for statistical analysis. *p* < .05 was regarded as the threshold of significance.

## 3 RESULTS

### 3.1 Circ_0138960 expression is elevated in patients with periodontal disease

**Figure 1:**
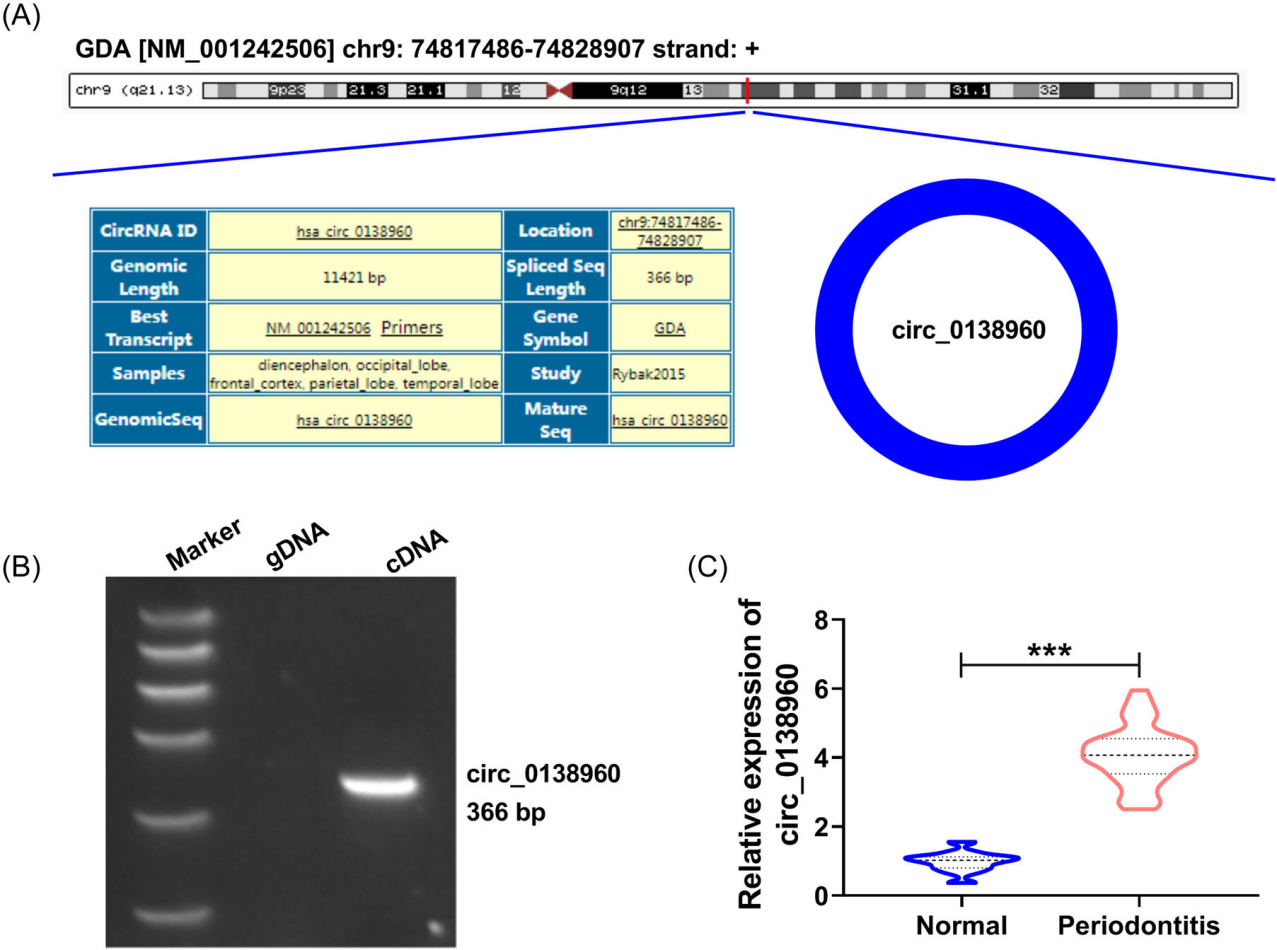
Circ_0138960 expression is elevated in the gingival tissues of patients with periodontal disease. (A) The information of circ_0138960 in Circinteractome database was shown. Circ_0138960 is derived from the back‐splicing of the GDA gene. (B) Circ_0138960 product amplified from gDNA or cDNA with divergent primers was examined using qPCR. (C) Circ_0138960 content in the gingival tissues of 37 patients with periodontal disease and 25 normal controls was determined using RT‐qPCR. ***p < .001. gDNA, genomic DNA; RT‐qPCR, reverse transcription‐quantitative polymerase chain reaction.

Circ_0138960 (366 nt) is derived from the back‐splicing of the gene GDA (Figure 1A). Meanwhile, our data displayed that circ_0138960 was only amplified using divergent primers in the cDNA group, rather than gDNA (Figure 1B). The expression of circ_0138960 in periodontitis was analyzed. Circ_0138960 was notably upregulated in patients with periodontal disease (*n* = 37) versus normal controls (*n* = 25) (Figure 1C, *p* < .001). To identify the association of circ_0138960 expression with the clinicopathologic characteristics, the 37 patients with periodontitis disease were then classified in Table 1. Result displayed that circ_0138960 expression was associated with Order of severity and Grade of periodontitis (*p* < .05). Overall, circ_0138960 was associated with periodontitis pathology.

### 3.2 LPS treatment suppresses PDLC proliferation and induces apoptosis and inflammation

**Figure 2:**
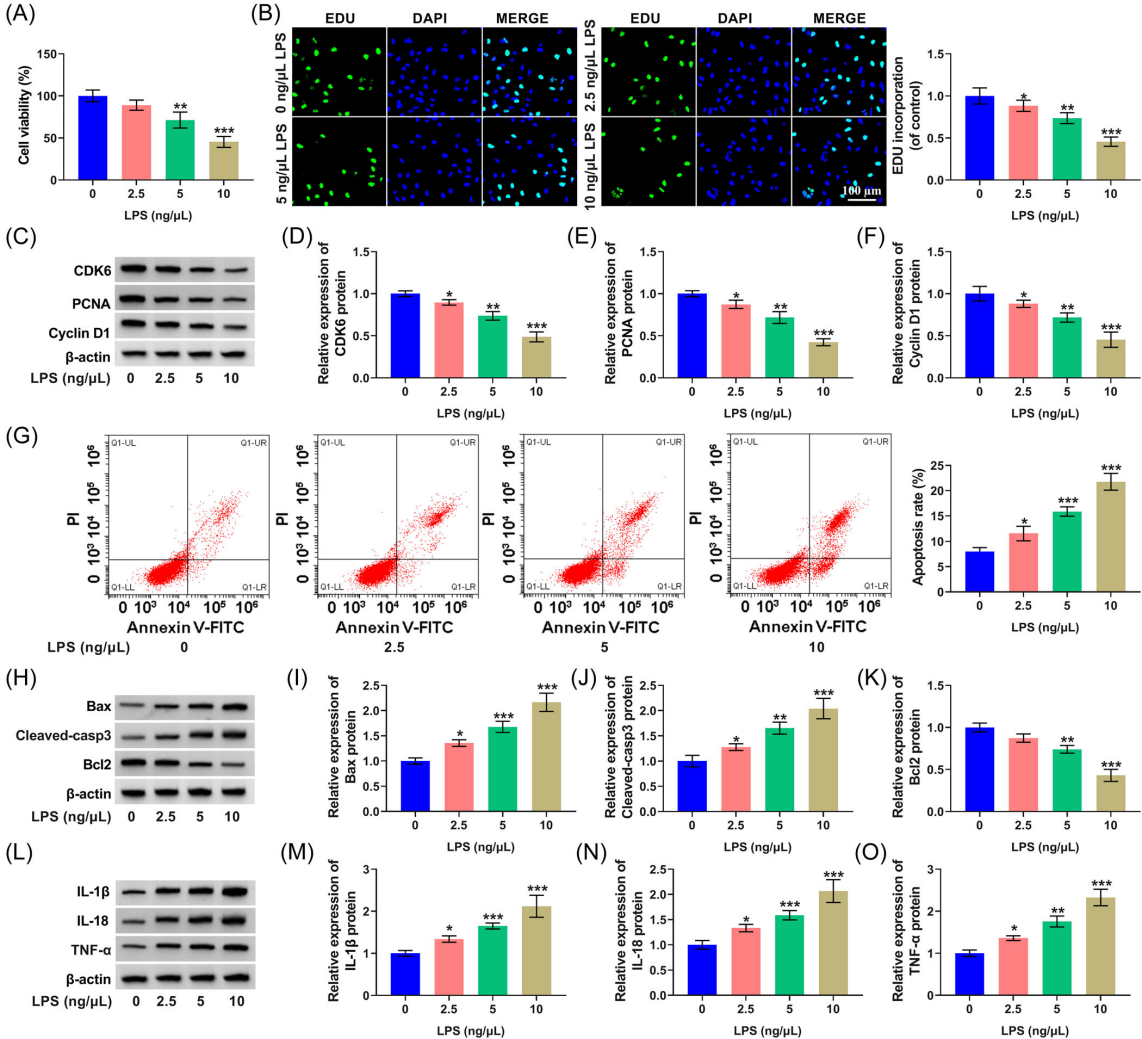
LPS treatment suppresses PDLC proliferation and induces apoptosis and inflammation. (A−O) PDLCs were stimulated with 2.5, 5, or 10 ng/μl LPS for 12 h. (A and B) Cell proliferation ability was assessed via CCK8 and EDU assay. (C−F) Three proliferation‐associated markers (CDK6, PCNA, and Cyclin D1) were measured using Western blot. (G) PDLC apoptosis was analyzed using flow cytometry. (H−K) Three apoptosis‐associated indicators (Bax, Cleaved‐casp3, and Bcl2) protein levels were detected by Western blot assay. (L−O) Three proinflammatory cytokines (IL‐1β, IL‐18, and TNF‐α) protein levels were determined. *p < .05, **p < .01, ***p < .001. CCK8, Cell Counting Kit; EDU, 5‐ethynyl‐20‐deoxyuridine; PCNA, proliferating cell nuclear antigen; PDLCs, periodontal ligament cells.

Furthermore, the periodontitis cell model was established by stimulating PDLCs with LPS at the dose of 2.5, 5, or 10 ng/μl for 12 h. We found that PDLC cell viability was reduced upon LPS treatment in a dose‐dependent manner (Figure 2A). LPS exposure gradually diminished PDLC proliferation, evidenced by a reduced percentage of EDU^+^ cells (Figure 2B). Three proliferation‐associated markers (CDK6, PCNA, and Cyclin D1) were downregulated in PDLCs by LPS stimulation in a concentration‐dependent way (Figure 2C−F). LPS exposure induced the apoptosis of PDLCs in a dose‐dependent manner (Figure 2G). Proapoptotic proteins (Bax and Cleaved‐casp3) were elevated via LPS stimulation, while Bcl2 (an antiapoptotic marker) exhibited an opposite effect (Figure 2H−K). LPS gradually induced the release of proinflammatory cytokines (IL‐1β, IL‐18, and TNF‐α) (Figure 2L−O). Overall, LPS suppressed PDLC proliferation and boosted cell apoptosis and inflammation in a dose‐dependent manner.

### 3.3 LPS‐induced dysfunction in PDLCs can be largely reversed by silencing circ_0138960

**Figure 3:**
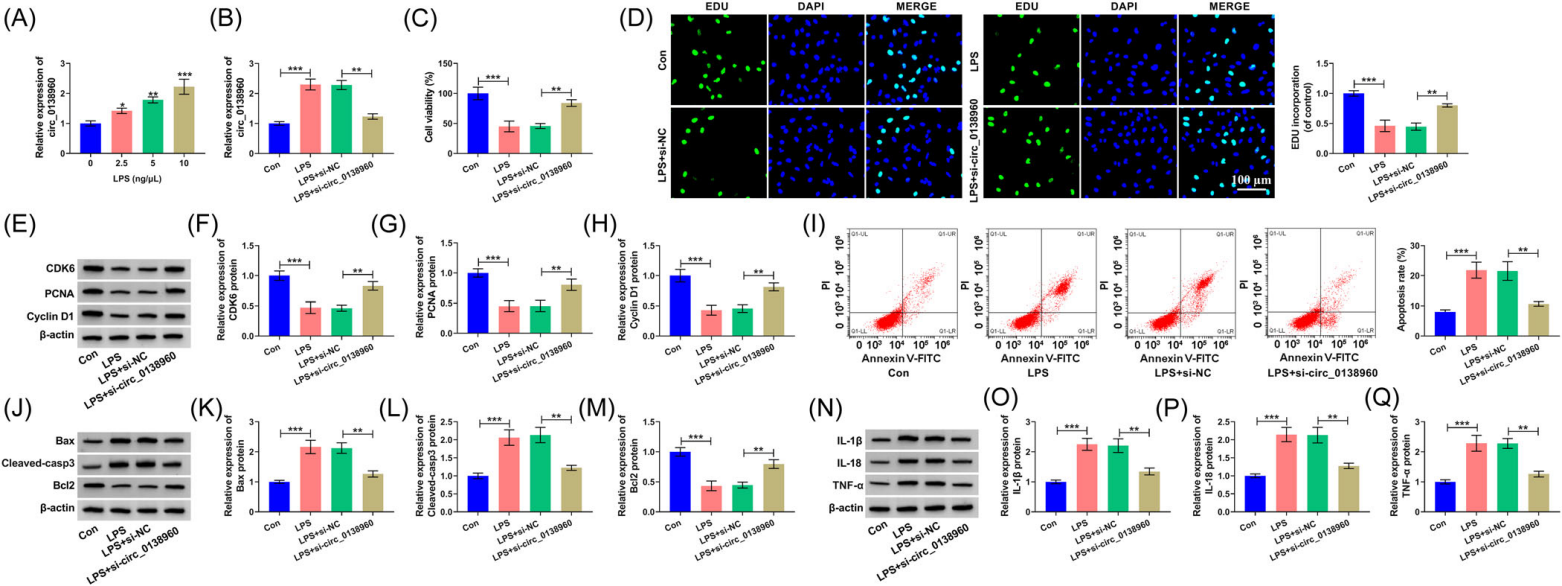
LPS‐induced dysfunction in PDLCs can be largely reversed by silencing circ_0138960. (A) PDLCs were stimulated with LPS at the dose of 2.5, 5, or 10 ng/μl for 12 h. Circ_0138960 content in treated PDLCs was assessed using RT‐qPCR. (B−Q) PDLCs treated with LPS (10 ng/μl; 12 h) were transfected with si‐NC or si‐circ_0138960. (B) RT‐qPCR analysis of circ_0138960 content. (C) Cell viability was analyzed by CCK8 assay. (D) Cell proliferation was analyzed by EDU assay. (E−H) The expression of CDK6, PCNA, and Cyclin D1 was determined by Western blot assay. (I) Apoptosis rate was assessed using flow cytometry. (J−M) Bax, Cleaved‐casp3, and Bcl2 protein levels were analyzed using Western blot. (N−Q) Western blot analysis of IL‐1β, IL‐18, and TNF‐α protein levels. *p < .05, **p < .01, ***p < .001. CCK8, Cell Counting Kit; EDU, 5‐ethynyl‐20‐deoxyuridine; LPS, lipopolysaccharide; PCNA, proliferating cell nuclear antigen; PDLCs, periodontal ligament cells; RT‐qPCR, reverse transcription‐quantitative polymerase chain reaction.

LPS improved circ_0138960 content in PDLCs in a dose‐dependent manner (Figure 3A). To analyze the biological role of circ_0138960 in LPS‐induced PDLCs, we performed loss‐of‐function experiments. LPS‐induced circ_0138960 enhancement in PDLCs was reversed via si‐circ_0138960 introduction (Figure 3B). LPS exposure dampened PDLC proliferative ability, which was rescued via si‐circ_0138960 (Figure 3C−H). LPS‐triggered PDLC apoptosis was largely offset via silencing circ_0138960 (Figure 3I−M). Beyond that, LPS‐mediated proinflammatory cytokines (IL‐1β, IL‐18, and TNF‐α) increasing were also effectively counteracted via circ_0138960 absence (Figure 3N−Q). Overall, circ_0138960 interference protected PDLCs from LPS‐induced dysfunction.

### 3.4 Circ_0138960 acts as a molecular sponge for miR‐518a‐5p

**Figure 4:**
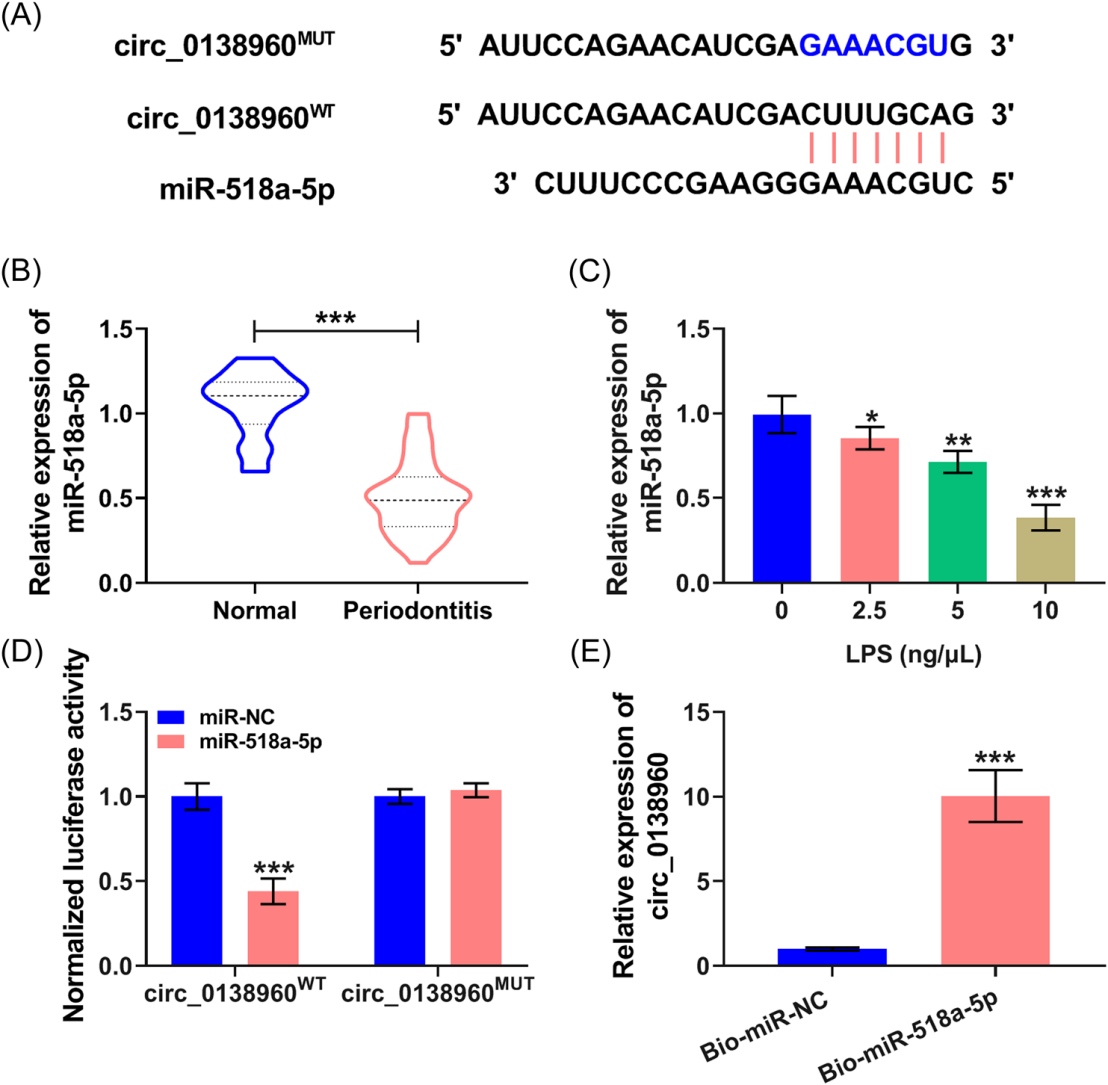
Circ_0138960 acts as a molecular sponge for miR‐518a‐5p. (A) Circinteractome database showed that circ_0138960 harbored the potential binding sites with miR‐518a‐5p. (B) RT‐qPCR was conducted to determine the expression of miR‐518a‐5p in the gingival tissues of patients with periodontal disease (*n* = 37) and normal controls (*n* = 25). (C) RT‐qPCR was conducted to examine the level of miR‐518a‐5p in PDLCs stimulated with LPS at the dose of 2.5, 5, or 10 ng/μl for 12 h. (D and E) The target relation between circ_0138960 and miR‐518a‐5p was confirmed by dual‐luciferase reporter assay and RNA‐pull down assay. *p < .05, **p < .01, ***p < .001. LPS, lipopolysaccharide; PDLCs, periodontal ligament cells; RT‐qPCR, reverse transcription‐quantitative polymerase chain reaction.

It is widely accepted that circRNAs can sponge miRNAs to regulate cell physiological and pathological processes.^12^ Using the Circinteractome database, all possible miRNA targets of circ_0138960 were predicted (Table 3), and four miRNAs with a high binding score of 99 were selected, including miR‐1205, miR‐1299, miR‐518a‐5p, and miR‐527. Subsequently, RNA‐pull down assay showed that miR‐518a‐5p and miR‐527 could be pulled down by the circ_0138960 probe, especially miR‐518a‐5p (Supporting Information: Figure 1A). As displayed in Figure 4A, putative binding sites were found between circ_0138960 and miR‐518a‐5p. We found that miR‐518a‐5p expression was prominently decreased in patients with periodontal disease (Figure 4B). LPS exposure reduced miR‐518a‐5p expression in PDLCs in a dose‐dependent manner (Figure 4C). Dual‐luciferase reporter assay exhibited that circ_0138960^WT^ luciferase activity was conspicuously dwindled via miR‐518a‐5p overexpression, while the mutant group (circ_0138960^MUT^) remained unchanged (Figure 4D). In parallel, circ_0138960 was pulled down when using Bio‐miR‐518a‐5p (Figure 4E). Overall, circ_0138960 sequesters miR‐518a‐5p in PDLCs.

### 3.5 Circ_0138960/miR‐518a‐5p regulated LPS‐induced PDLC injury

**Figure 5:**
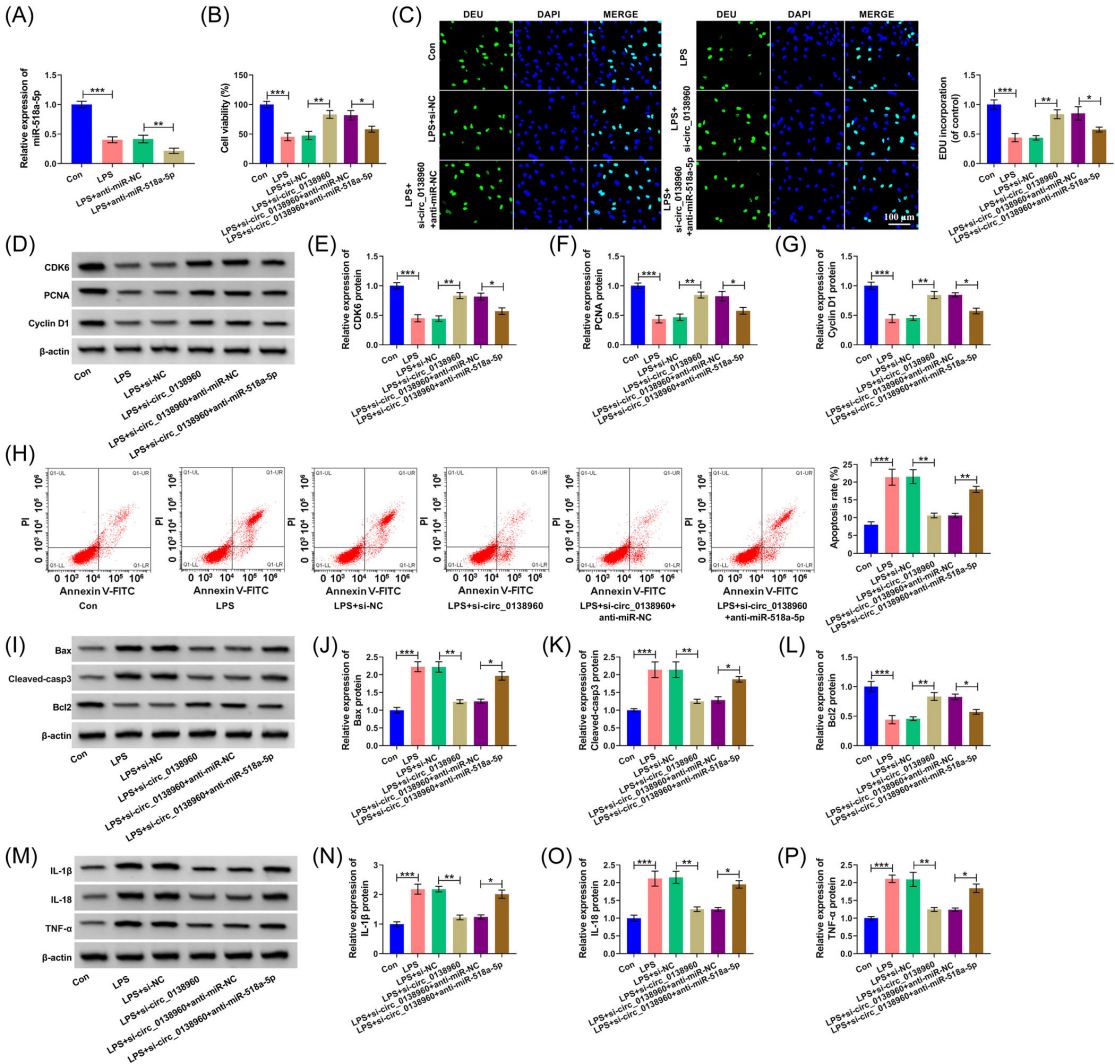
Circ_0138960 absence‐mediated protective effects in LPS‐induced PDLCs are largely overturned by silencing miR‐518a‐5p. (A) The expression of miR‐518a‐5p was examined via RT‐qPCR in PDLCs in the following four groups: Control, LPS, LPS + anti‐miR‐NC, and LPS + anti‐miR‐518a‐5p. (B−P) PDLCs were divided into the following six groups: Control, LPS, LPS + si‐NC, LPS + si‐circ_0138960, LPS + si‐circ_0138960 + anti‐miR‐NC, and LPS + si‐circ_0138960 + anti‐miR‐518a‐5p. (B) CCK8 assay was performed to analyze the viability of PDLCs. (C) Cell proliferation was assessed via EDU. (D−G) CDK6, PCNA, and Cyclin D1 protein levels were analyzed using Western blot. (H) Flow cytometry was conducted to evaluate cell apoptosis. (I−L) Western blot assay was carried out to detect the expression of Bax, Cleaved‐casp3, and Bcl2. (M−P) Western blot assay was conducted to detect IL‐1β, IL‐18, and TNF‐α protein levels. *p < .05, **p < .01, ***p < .001. CCK8, Cell Counting Kit; EDU, 5‐ethynyl‐20‐deoxyuridine; LPS, lipopolysaccharide; PCNA, proliferating cell nuclear antigen; PDLCs, periodontal ligament cells.

Knockdown efficiency of anti‐miR‐518a‐5p in PDLCs was validated (Figure 5A). The silence of miR‐518a‐5p reduced PDLC viability and suppressed proliferation again (Figure 5B−G). In addition, we found that anti‐miR‐518a‐5p induced apoptosis and proinflammatory cytokines (IL‐1β, IL‐18, and TNF‐α) (Figure 5H−P). These findings together demonstrated that circ_0138960 absence attenuated LPS‐induced PDLC dysfunction partly via miR‐518a‐5p.

### 3.6 HDAC6 is a target of miR‐518a‐5p

**Figure 6:**
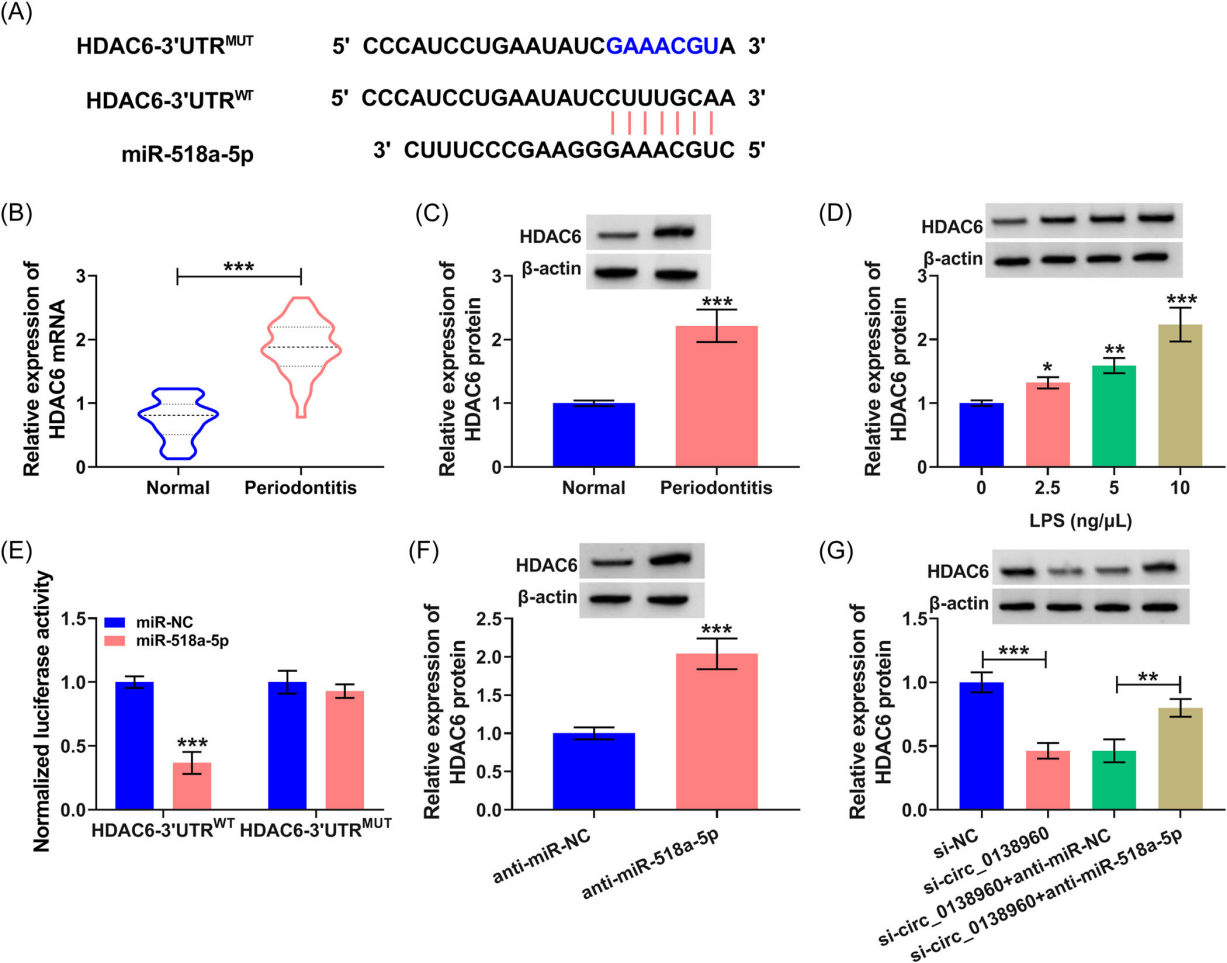
HDAC6 is a target of miR‐518a‐5p. (A) Bioinformatics database targetscan predicted the possible binding sequence between miR‐518a‐5p and HDAC6. (B and C) The mRNA and protein levels of HDAC6 were determined in the gingival tissues of patients with periodontal disease and normal controls by RT‐qPCR and Western blot assay. (D) Western blot assay was conducted to measure the protein level of HDAC6 in PDLCs stimulated with LPS at the dose of 2.5, 5, or 10 ng/μl for 12 h. (E) Their binding was verified by dual‐luciferase reporter assay. (F) Western blot assay was performed to determine the protein level of HDAC6 in PDLCs transfected with anti‐miR‐NC or anti‐miR‐518a‐5p. (G) Western blot analysis of effects of si‐circ_0138960 and anti‐miR‐518a‐5p on HDAC6 protein level. *p < .05, **p < .01, ***p < .001. HDAC6, histone deacetylase 6; mRNA, messenger RNA; PDLCs, periodontal ligament cells; RT‐qPCR, reverse transcription‐quantitative polymerase chain reaction.

We predicted the interacted mRNAs of miR‐518a‐5p using the targetscan database. Among them, five mRNAs that play regulatory roles in periodontitis pathology, including PTEN,^13^ SOCS6,^14^, ^15^ HDAC6,^16^ NR2F2,^17^ and RORA.^18^, ^19^ We analyzed the expression of these five mRNAs in miR‐518a‐5p‐silenced PDLCs and found that SOCS6 and HDAC6 could be negatively regulated by miR‐518a‐5p, especially HDAC6 (Supporting Information: Figure 1B). Hence, HDAC6 was selected as the follow‐up subject. Their putative binding sequence was exhibited in Figure 6A. HDAC6 expression was upregulated in the gingival tissues of patients with periodontal disease (Figures 6B,C). LPS exposure upregulated the protein level of HDAC6 in PDLCs in a dose‐dependent manner (Figure 6D). The luciferase intensity of HDAC6‐3'UTR^WT^ was conspicuously reduced via miR‐518a‐5p overexpression (Figure 6E), rather than mutant group (Figure 6E), indicating their direct interaction. MiR‐518a‐5p interference augmented HDAC6 protein level in PDLCs (Figure 6F). Circ_0138960 absence reduced HDAC6 content in PDLCs, and anti‐miR‐518a‐5p largely recovered the phenomenon (Figure 6G), suggesting that circ_0138960 could act as a miR‐518a‐5p sponge to positively regulate HDAC6 content in PDLCs.

### 3.7 HDAC6 overexpression reverses circ_0138960 interference‐mediated impacts in PDLCs upon LPS treatment

We analyzed the biological role of HDAC6 in LPS‐induced PDLCs by loss‐of‐function experiments. Knockdown efficiency of si‐HDAC6 in PDLCs was validated (Supporting Information: Figure 2A). LPS‐induced PDLC dysfunction was effectively diminished via silencing HDAC6 (Supporting Information: Figure 2B–2Q), indicating that HDAC6 knockdown protected PDLCs from LPS‐induced dysfunction.

**Figure 7:**
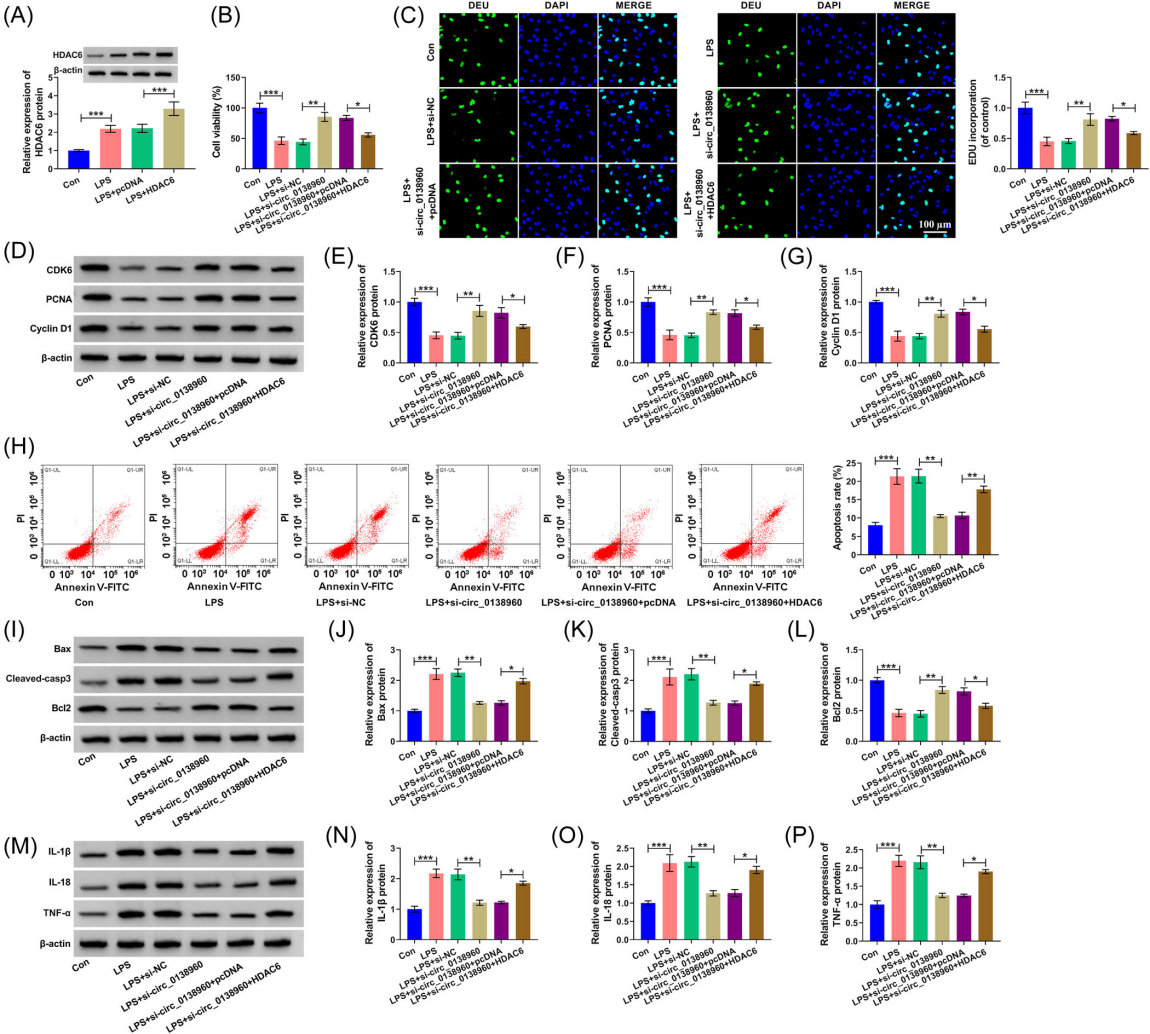
HDAC6 overexpression reverses circ_0138960 interference‐mediated impacts in PDLCs upon LPS treatment. (A) Western blot assay was conducted to measure the protein level of HDAC6 in PDLCs in the following four groups: Control, LPS, LPS + pcDNA, and LPS + HDAC6. (B and C) CCK8 and EDU assay assess cell proliferative ability. (D−G) CDK6, PCNA, and Cyclin D1 protein expression were determined via western blot. (H) PDLC apoptosis rate was analyzed via flow cytometry. (I−L) Bax, Cleaved‐casp3, and Bcl2 protein levels were examined via western blot. (M−P) Western blot analysis of IL‐1β, IL‐18, and TNF‐α protein levels. *p < .05, **p < .01, ***p < .001. CCK8, Cell Counting Kit; EDU, 5‐ethynyl‐20‐deoxyuridine; HDAC6, histone deacetylase 6; LPS, lipopolysaccharide; pcDNA, plasmid cloning DNA; PCNA, proliferating cell nuclear antigen; PDLCs, periodontal ligament cells.

Western blot assay confirmed the overexpression efficiency of HDAC6 plasmid in PDLCs (Figure 7A). The protective action of cell viability and proliferation of LPS‐induced PDLCs caused by circ_0138960 depletion was reversed by the overexpression of HDAC6 (Figure 7B−G). Circ_0138960 silencing suppressed LPS‐induced apoptosis in PDLCs, and the addition of HDAC6 plasmid triggered cell apoptosis again (Figure 7H−L). The addition of HDAC6 plasmid also induced the inflammation of PDLCs (Figure 7M−P). Taken together, a protective role of circ_0138960 absence on LPS‐induced PDLCs was partly mitigated via HDAC6 downregulating.

## 4 DISCUSSION

Inflammation is considered to be a major factor in periodontitis pathology. Therefore, alleviating inflammation‐induced PDLCs injury is important for the treatment of periodontitis.^20^ LPS is widely utilized in inflammation‐associated cell models in vitro.^21^ In this study, LPS‐induced PDLCs were used as a periodontitis cell model in vitro. Our study confirmed that LPS stimulation reduced PDLC proliferation and induced apoptosis and inflammation in a dose‐dependent manner. Noncoding RNAs, including circRNAs, are identified as promising targets for inflammation‐associated diseases such as periodontitis.^7^, ^22^ Here, we found that circ_0138960 was upregulated in patients with periodontal disease, which was consistent with a previous study.^9^ LPS exposure gradually upregulated circ_0138960 content in PDLCs. In addition, we found that circ_0138960 depletion relieved LPS‐induced PDLC dysfunction.

Accumulating evidence has suggested that circRNAs play important regulatory roles in multiple human diseases by acting as miRNA sponges.^23^, ^24^ For instance, circ_0003204 restrains endothelial cell growth, migration, and angiogenesis in atherosclerosis by sponging miR‐370‐3p.^25^ Serving as a miR‐31‐5p sponge, circ‐BPTF might aggravate bladder cancer development.^26^ Using bioinformatics analysis, miR‐518a‐5p appeared as a circ_0138960 target in PDLCs. miR‐518a‐5p expression was significantly reduced in patients with periodontal disease. Meanwhile, we uncovered that LPS treatment decreased the miR‐518a‐5p level in PDLCs in a dose‐dependent manner. A previous study reported that lncRNA 01126 contributes to periodontitis pathogenesis by sponging miR‐518a‐5p,^27^ indicating that miR‐518a‐5p played a protective role in periodontitis. We found miR‐518a‐5p downregulation might reverse circ_0138960 absence‐provoked protective action in LPS‐stimulated PDLCs, indicating that circ_0138960 silencing protected PDLCs against LPS‐caused damage largely via miR‐518a‐5p.

It is well established that miRNAs can accomplish their biological function via interacting with mRNAs.^28^, ^29^ Our findings uncovered that miR‐518a‐5p is directly bound to the 3'UTR of HDAC6 in PDLCs. HDAC6 is a member of the histone deacetylase family, and it regulates multiple cellular processes through its histone deacetylase activity.^30^ A previous study found that IL‐1β elevates the mRNA expression of HDAC6 in periodontal ligament fibroblasts,^16^ implying its pivotal role in periodontitis pathology. We confirmed that HDAC6 was elevated in periodontitis sufferers and LPS‐stimulated PDLCs. In addition, we uncovered circ_0138960 positively modulated HDAC6 content via sponging miR‐518a‐5p in PDLCs. HDAC6 knockdown protected PDLCs from LPS‐induced dysfunction. Beyond that, compensation experiments disclosed that circ_0138960 silencing‐induced protective influences in LPS‐stimulated PDLCs were largely offset by HDAC6 accumulation, suggesting that circ_0138960 interference played protective impacts in LPS‐stimulated PDLCs largely by downregulating HDAC6.

**Figure 8:**
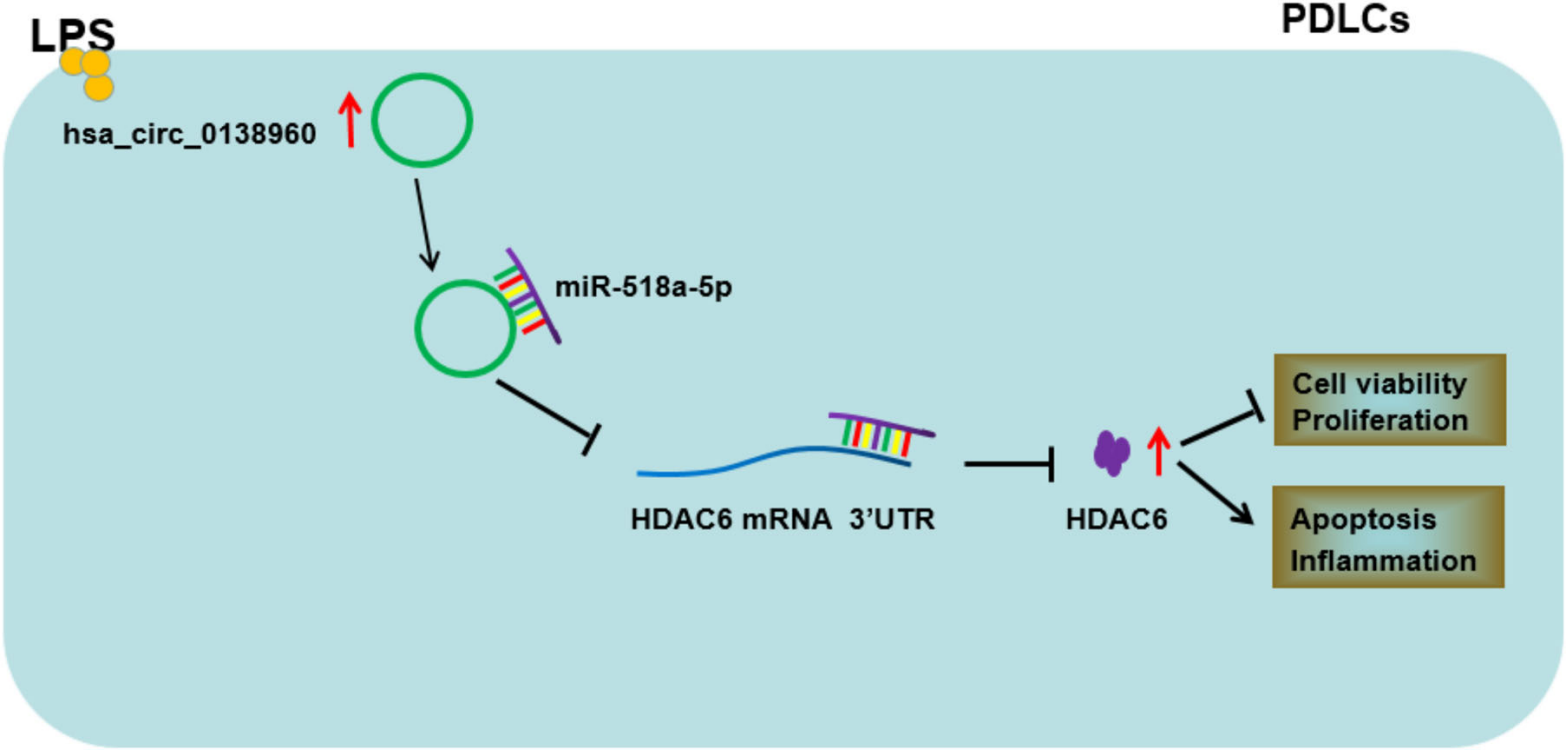
Schematic diagram reveals circ_0138960/miR‐518a‐5p/HDAC6 axis in LPS‐induced PDLCs. HDAC6, histone deacetylase 6; LPS, lipopolysaccharide; PDLCs, periodontal ligament cells.

In summary, our findings confirmed that circ_0138960 contributed to LPS‐induced dysfunction in PDLCs by miR‐518a‐5p/HDAC6 (Figure 8). This new ceRNA mechanism provides novel potential therapeutic targets for periodontitis. However, the current research is limited by the small sample size and the lack of animal model experiments. Hence, our future research will try to include more patients and construct periodontitis animal models to further verify our conclusions. Furthermore, the working mechanism of HDAC6 in periodontitis pathology still needs to be explored.

## Supporting information


**Supplementary Figure 1. The candidate miRNA targets circ_0138960 and the candidate mRNA targets miR‐518a‐5p**. (A) A RNA‐pull down assay using a biotin‐labeled circ_0138960 probe was conducted to analyze the interaction between circ_0138960 and four possible miRNA targets, including miR‐1205, miR‐1299, miR‐518a‐5p, and miR‐527. (B) RT‐qPCR analysis of the effects of anti‐miR‐518a‐5p on five possible mRNA targets levels (PTEN, SOCS6, HDAC6, NR2F2, and RORA) in PDLCs transfected with anti‐miR‐NC or anti‐miR‐518a‐5p. *P<0.05, **P<0.01, ***P<0.001.

**Supplementary Figure 2. HDAC6 depletion protects PDLCs from LPS‐induced dysfunction**. (A‐Q) PDLCs treated with LPS (10 ng/μL; 12 h) were transfected with si‐NC or si‐HDAC6. (A) HDAC6 protein expression in PDLCs was measured using western blot. (B) CCK‐8 analysis of cell viability. (C and D) EDU analysis of PDLC proliferation ability. (E‐H) CDK6, PCNA, and Cyclin D1 protein levels were examined using western blot. (I) Flow cytometry analysis of apoptosis rate. (J‐M) Bax, Cleaved‐casp3, and Bcl2 protein expression were measured by Western blot assay. (N‐Q) Western blot assay was carried out to measure the protein levels of IL‐1β, IL‐18, and TNF‐α. **P<0.01, ***P<0.001.

## References

[1] Slots J . Periodontitis: facts, fallacies and the future. Periodontol 2000. 2017;75(1):7‐23. 10.1111/prd.12221 28758294

[2] Bartold PM . Lifestyle and periodontitis: the emergence of personalized periodontics. Periodontol 2000. 2018;78(1):7‐11. 10.1111/prd.12237 30198129

[3] Choi GE , Hyun KY . Inhibitory effect of Acer tegmentosum maxim extracts on P. gingivalis LPS‐induced periodontitis. Arch Oral Biol. 2020;109:104529. 10.1016/j.archoralbio.2019.104529 31574324

[4] Han Y , Wang F , Shao L , Huang P , Xu Y . LncRNA TUG1 mediates lipopolysaccharide‐induced proliferative inhibition and apoptosis of human periodontal ligament cells by sponging miR‐132. Acta Biochim Biophys Sin (Shanghai). 2019;51(12):1208‐1215. 10.1093/abbs/gmz125 31735958

[5] Wang F , Chen X , Han Y , Xi S , Wu G . circRNA CDR1as regulated the proliferation of human periodontal ligament stem cells under a Lipopolysaccharide‐Induced inflammatory condition. Mediators Inflamm. 2019;2019:1625381. 10.1155/2019/1625381 31582895 PMC6754938

[6] Zhang Z , Yang T , Xiao J . Circular RNAs: promising biomarkers for human diseases. EBioMedicine. 2018;34:267‐274. 10.1016/j.ebiom.2018.07.036 30078734 PMC6116471

[7] Jiao K , Walsh LJ , Ivanovski S , Han P . The emerging regulatory role of circular RNAs in periodontal tissues and cells. Int J Mol Sci. 2021;22(9):4636. 10.3390/ijms22094636 33924932 PMC8124626

[8] Wu T , Sun Y , Sun Z , et al. Hsa_circ_0042823 accelerates cancer progression via miR‐877‐5p/FOXM1 axis in laryngeal squamous cell carcinoma. Ann Med. 2021;53(1):960‐970. 10.1080/07853890.2021.1934725 34124974 PMC8204964

[9] Li J , Xie R . Circular RNA expression profile in gingival tissues identifies circ_0062491 and circ_0095812 as potential treatment targets. J Cell Biochem. 2019;120(9):14867‐14874. 10.1002/jcb.28748 31021476

[10] Panda AC . Circular RNAs Act as miRNA sponges. Adv Exp Med Biol. 2018;1087:67‐79. 10.1007/978-981-13-1426-1_6 30259358

[11] Thomson DW , Dinger ME . Endogenous microRNA sponges: evidence and controversy. Nat Rev Genet. 2016;17(5):272‐283. 10.1038/nrg.2016.20 27040487

[12] Hansen TB , Jensen TI , Clausen BH , et al. Natural RNA circles function as efficient microRNA sponges. Nature. 2013;495(7441):384‐388. 10.1038/nature11993 23446346

[13] Fu C , Wei Z , Zhang D . PTEN inhibits inflammatory bone loss in Ligature‐Induced periodontitis via IL1 and TNF‐α. BioMed Res Int. 2019;2019:6712591. 10.1155/2019/6712591 31886238 PMC6914910

[14] Du W , Wang L , Liao Z , Wang J . Circ_0085289 alleviates the progression of periodontitis by regulating let‐7f‐5p/SOCS6 pathway. Inflammation. 2021;44(4):1607‐1619. 10.1007/s10753-021-01445-8 33710445

[15] Chen H , Lan Z , Li Q , Li Y . Abnormal expression of long noncoding RNA FGD5‐AS1 affects the development of periodontitis through regulating miR‐142‐3p/SOCS6/NF‐κB pathway. Artif Cells, Nanomed, Biotechnol. 2019;47(1):2098‐2106. 10.1080/21691401.2019.1620256 31144533

[16] Memmert S , Nogueira AVB , Damanaki A , et al. Damage‐regulated autophagy modulator 1 in oral inflammation and infection. Clin Oral Investig. 2018;22(8):2933‐2941. 10.1007/s00784-018-2381-6 29442188

[17] Li W , Zhang Z , Li Y , Wang Z . Abnormal hsa_circ_0003948 expression affects chronic periodontitis development by regulating miR‐144‐3p/NR2F2/PTEN signaling. J Periodont Res. 2021;57:316‐323. 10.1111/jre.12961 34910830

[18] Wang J , Du C , Xu L . Circ_0081572 inhibits the progression of periodontitis through regulating the miR‐378h/RORA axis. Arch Oral Biol. 2021;124:105053. 10.1016/j.archoralbio.2021.105053 33524877

[19] Huang N , Li C , Sun W , Wu J , Xiao F . Long non‐coding RNA TUG1 participates in LPS‐induced periodontitis by regulating miR‐498/RORA pathway. Oral Dis. 2021;27(3):600‐610. 10.1111/odi.13590 32762066

[20] Yoshihara‐Hirata C , Yamashiro K , Yamamoto T , et al. Anti‐HMGB1 neutralizing antibody attenuates periodontal inflammation and bone resorption in a murine periodontitis model. Infect Immun. 2018;86(5):e00111‐18. 10.1128/iai.00111-18 29531138 PMC5913859

[21] Wilson M . Biological activities of lipopolysaccharides from oral bacteria and their relevance to the pathogenesis of chronic periodontitis. Sci Prog. 1995;78(Pt 1):19‐34. 7597416

[22] Marques‐Rocha JL , Samblas M , Milagro FI , Bressan J , Martínez JA , Marti A . Noncoding RNAs, cytokines, and inflammation‐related diseases. FASEB J. 2015;29(9):3595‐3611. 10.1096/fj.14-260323 26065857

[23] Tay Y , Rinn J , Pandolfi PP . The multilayered complexity of ceRNA crosstalk and competition. Nature. 2014;505(7483):344‐352. 10.1038/nature12986 24429633 PMC4113481

[24] Hsiao KY , Sun HS , Tsai SJ . Circular RNA—new member of noncoding RNA with novel functions. Exp Biol Med (Maywood). 2017;242(11):1136‐1141. 10.1177/1535370217708978 28485684 PMC5478007

[25] Zhang S , Song G , Yuan J , et al. Circular RNA circ_0003204 inhibits proliferation, migration and tube formation of endothelial cell in atherosclerosis via miR‐370‐3p/TGFβR2/phosph‐SMAD3 axis. J Biomed Sci. 2020;27(1):11. 10.1186/s12929-019-0595-9 31900142 PMC6941276

[26] Bi J , Liu H , Cai Z , et al. Circ‐BPTF promotes bladder cancer progression and recurrence through the miR‐31‐5p/RAB27A axis. Aging. 2018;10(8):1964‐1976. 10.18632/aging.101520 30103209 PMC6128440

[27] Zhou M , Hu H , Han Y , et al. Long non‐coding RNA 01126 promotes periodontitis pathogenesis of human periodontal ligament cells via miR‐518a‐5p/HIF‐1α/MAPK pathway. Cell Prolif. 2021;54(1):e12957. 10.1111/cpr.12957 33231338 PMC7791173

[28] Fabian MR , Sonenberg N , Filipowicz W . Regulation of mRNA translation and stability by microRNAs. Annu Rev Biochem. 2010;79:351‐379. 10.1146/annurev-biochem-060308-103103 20533884

[29] O'Brien J , Hayder H , Zayed Y , Peng C . Overview of MicroRNA biogenesis, mechanisms of actions, and circulation. Front Endocrinol. 2018;9:402. 10.3389/fendo.2018.00402 PMC6085463 30123182

[30] Yan GQ , Wang X , Yang F , et al. MicroRNA‐22 promoted osteogenic differentiation of human periodontal ligament stem cells by targeting HDAC6. J Cell Biochem. 2017;118(7):1653‐1658. 10.1002/jcb.25931 28195408

