# Supplementary figures and images for: Circ_0138960 contributes to lipopolysaccharide‐induced periodontal ligament cell dysfunction
Source: Immun Inflamm Dis. 2022 Nov 7;10(12):e732. doi: 10.1002/iid3.732 (PMC9639461; doi:10.1002/iid3.732)

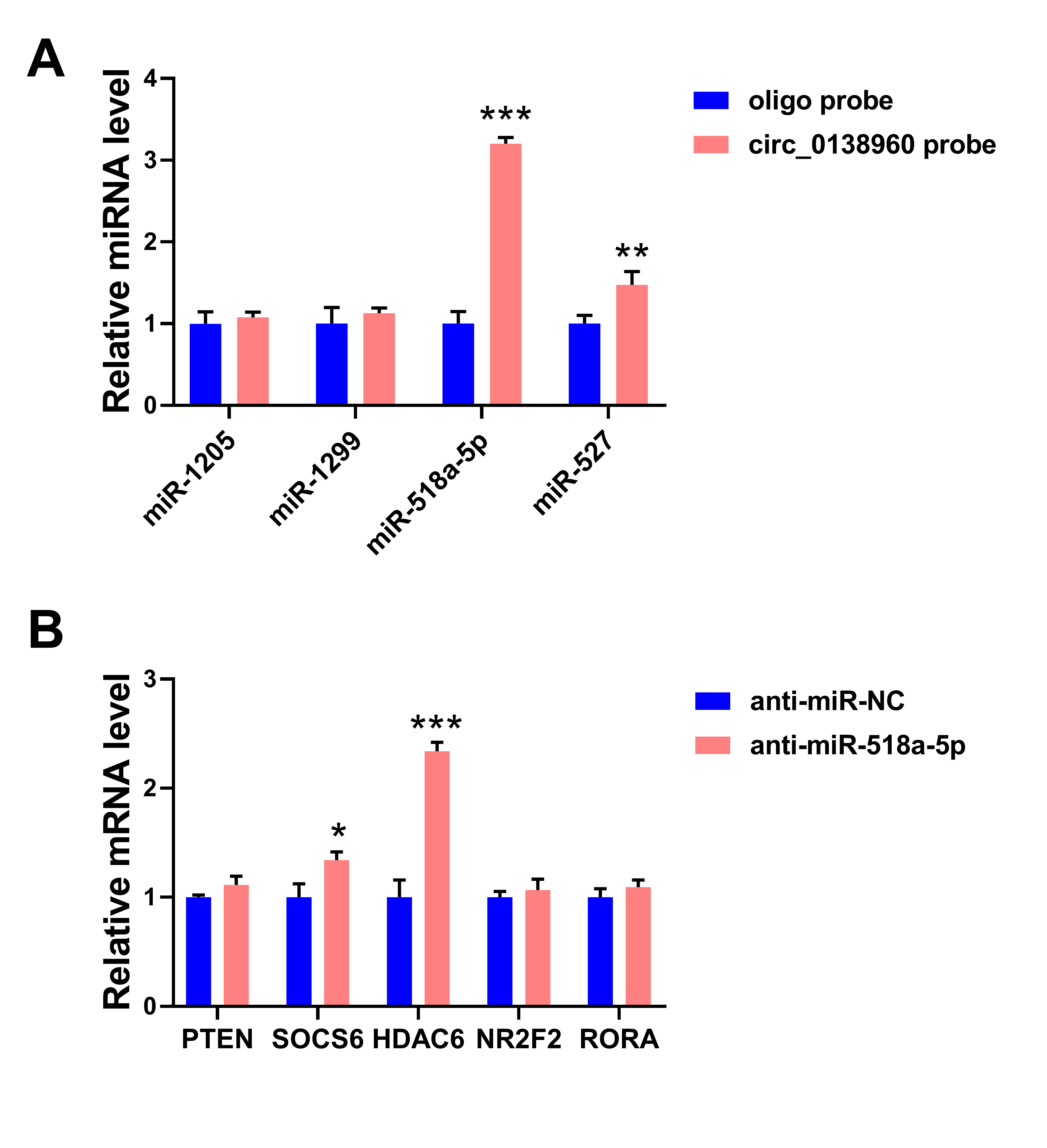

Supplement: Supplementary file 1 — Supplementary Figure 1. The candidate miRNA targets circ_0138960 and the candidate mRNA targets miR‐518a‐5p. (A) A RNA‐pull down assay using a biotin‐labeled circ_0138960 probe was conducted to analyze the interaction between circ_0138960 and four possible miRNA targets, including miR‐1205, miR‐1299, miR‐518a‐5p, and miR‐527. (B) RT‐qPCR analysis of the effects of anti‐miR‐518a‐5p on five possible mRNA targets levels (PTEN, SOCS6, HDAC6, NR2F2, and RORA) in PDLCs transfected with anti‐miR‐NC or anti‐miR‐518a‐5p. *P<0.05, **P<0.01, ***P<0.001. [file IID3-10-e732-s001.tif]

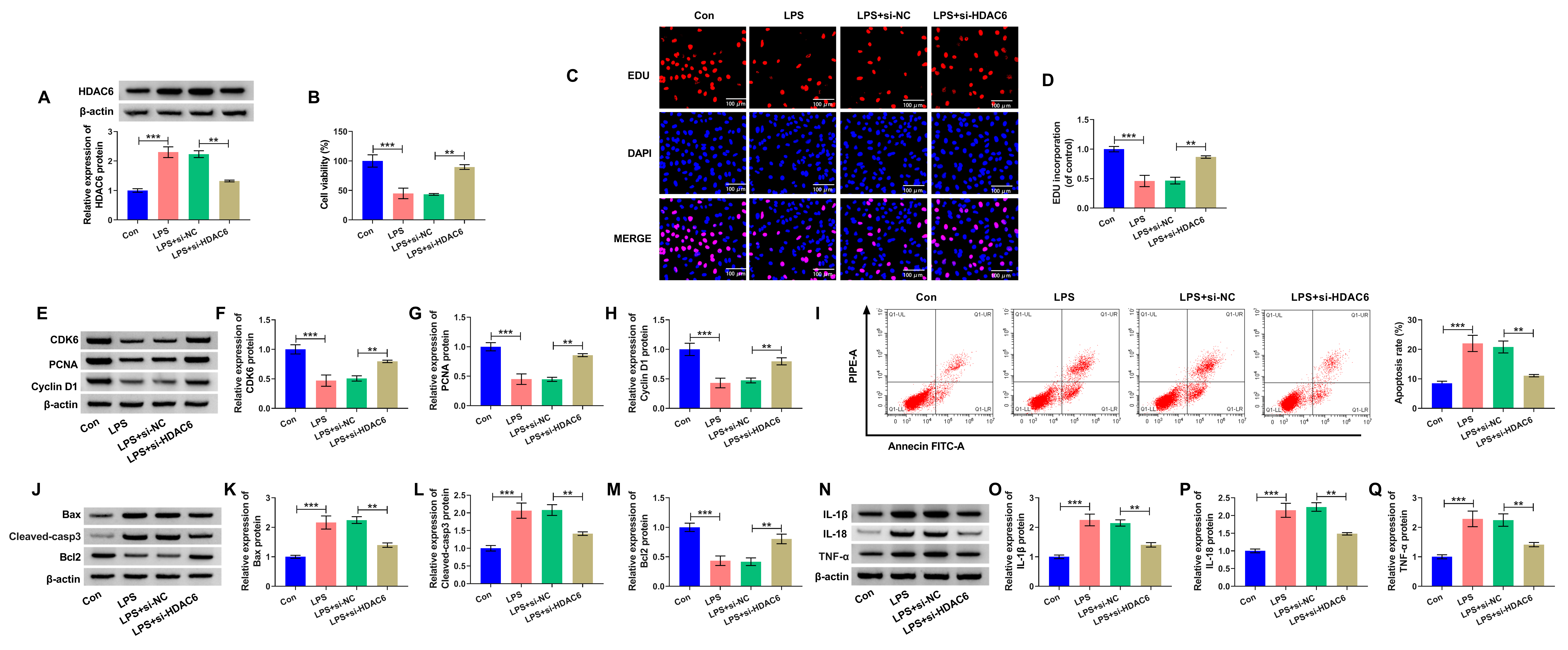

Supplement: Supplementary file 2 — Supplementary Figure 2. HDAC6 depletion protects PDLCs from LPS‐induced dysfunction. (A‐Q) PDLCs treated with LPS (10 ng/μL; 12 h) were transfected with si‐NC or si‐HDAC6. (A) HDAC6 protein expression in PDLCs was measured using western blot. (B) CCK‐8 analysis of cell viability. (C and D) EDU analysis of PDLC proliferation ability. (E‐H) CDK6, PCNA, and Cyclin D1 protein levels were examined using western blot. (I) Flow cytometry analysis of apoptosis rate. (J‐M) Bax, Cleaved‐casp3, and Bcl2 protein expression were measured by Western blot assay. (N‐Q) Western blot assay was carried out to measure the protein levels of IL‐1β, IL‐18, and TNF‐α. **P<0.01, ***P<0.001. [file IID3-10-e732-s002.tif]
